# Migration and Health: Freedom of Movement and Social Benefits for Chinese Migrant Workers

**DOI:** 10.1093/geroni/igab046.1878

**Published:** 2021-12-17

**Authors:** Fengxian Qiu, Heying Zhan, Jing Liu

**Affiliations:** 1 Anhui Normal University, Wuhu, Anhui, China (People's Republic); 2 Georgia State University, Georgia State University, Georgia, United States; 3 Zhejiang University of Finance and Economics, Hangzhou, Zhejiang, China (People's Republic)

## Abstract

Nearly 30% of China’s workforce consists of China’s rural-to-urban migrant workers, accounting for nearly 300 million of China’s population. Even though they have gained freedom of movement since the 1980s, they still have no access to healthcare in urban areas where they work. This study utilizes a mixed method of a survey with a sample of migrant workers from three Chinese emigration provinces (n=817) in 2018 and follow-up interviews with 30 migrant workers in 2020 to examine factors of migration experience affecting migrant worker’s health and healthcare. Using binary logistic regression, we found that migrant workers’ longer work experience is correlated with poorer self-rated health, their better financial status and level of hopefulness towards the future are positively correlated to self-rated health. Qualitative findings shed light on the cumulative effect of the length of work experience and fear of medical cost on migrant workers’ declining health. The lack of portability in health insurance and different reimbursement rates in health care access are structural barriers in health-seeking behaviors among migrant workers. Policy implications are presented in the global context of social rights and freedom of movement.

